# In Situ Determination of pH at Nanostructured Carbon Electrodes Using IR Spectroscopy

**DOI:** 10.3390/ma12244044

**Published:** 2019-12-05

**Authors:** Lolade Bamgbelu, Katherine B Holt

**Affiliations:** Department of Chemistry, University College London, 20 Gordon St, London WC1H 0AJ, UK; lolade.bamgbelu.14@ucl.ac.uk

**Keywords:** electrode, interface, spectroelectrochemistry, IR spectroscopy, pH, phosphate, carbon nanotubes

## Abstract

Changes in pH at electrode surfaces can occur when redox reactions involving the production or consumption of protons take place. Many redox reactions of biological or analytical importance are proton-coupled, resulting in localized interfacial pH changes as the reaction proceeds. Other important electrochemical reactions, such as hydrogen and oxygen evolution reactions, can likewise result in pH changes near the electrode. However, it is very difficult to measure pH changes located within around 100 µm of the electrode surface. This paper describes the use of in situ attenuated total reflectance (ATR) infrared (IR) spectroscopy to determine the pH of different solutions directly at the electrode interface, while a potential is applied. Changes in the distinctive IR bands of solution phosphate species are used as an indicator of pH change, given that the protonation state of the phosphate ions is pH-dependent. We found that the pH at the surface of an electrode modified with carbon nanotubes can increase from 4.5 to 11 during the hydrogen evolution reaction, even in buffered solutions. The local pH change accompanying the hydroquinone–quinone redox reaction is also determined.

## 1. Introduction

Carbon nanostructured electrodes are ubiquitous in electrochemistry, used as electrocatalyst supports [1], as energy storage electrodes with a large surface area, found in supercapacitors [2], as redox flow batteries [3], and in sensing and electroanalysis [4,5,6]. The biocompatible nature of some nanocarbons, such as carbon nanotubes, has led to their use in the modification of electrodes for biological sensing [7], the detection of neurotransmitters [8], the stimulation of nervous tissues [9], pathogen detection [10], and interfacing with enzymes [11]. However, in many key redox reactions of biological interest, such as the oxidation of dopamine and other quinones, the proton-coupled nature of the electron transfer can result in a local pH change [12]. In addition, relatively high potentials are applied to carbon electrodes during in vivo neural stimulation, and these may be sufficient to oxidize or reduce water—reactions that liberate or consume protons and hence change the local pH. Such pH changes can perturb redox equilibria or even damage tissue or enzymes in the case of embedded electrodes or biosensors. It is therefore of interest to develop a method to examine the pH in the region close to the electrode surface. This paper describes how in situ infrared (IR) spectroscopy can reveal significant pH changes at carbon electrode interfaces during operation. 

Previous attempts to measure pH at electrodes have required invasive measurement and specific electrode materials and architecture. Moreover, they could only probe narrow pH ranges [13]. For example, a form of previously used invasive sampling consisted of taking small volumes of solution from the interface or freezing them for later pH determination [14]. Mesh-capped pH probes have been used to measure pH at the mesh electrode, but this is a complex setup, and the electrode material is confined to metal meshes, such as nickel and platinum [15]. Rotating ring-disk electrodes can also be used, but investigations require specific electrode constructions [16]. Microelectrodes of pH-sensitive metal oxides (e.g., antimony oxides), which can be kept close to the surface of an electrode, have been used for scanning electrochemical microscopy measurements of electrode surface pH. However, such probes need careful calibration before use [17]. Optical techniques, for example combing confocal microscopy with pH-sensitive fluorophores, have also been used as pH probes for the electrode interface, but this approach requires complex instrumentation and is limited by the pH sensitivity range of the fluorescence probes [18]. There have also been previous reports using vibrational spectroscopy with “reporter” ions, as described in this paper. Raman spectroscopy was used to show changes in the concentration of ClO_4_^−^ in electrodes during methanol oxidation and oxygen reduction reactions [19]. Changes in the IR spectra of phosphate ions, as a function of applied potential, were used to show pH changes at a conducting ZnSe attenuated total reflectance (ATR) prism [20]. This approach allowed very subtle pH changes to be determined [21] but is inflexible in its requirement of a conducting ATR prism. Using a more versatile ATR IR spectroelectrochemical cell arrangement, with a working electrode located directly above a non-conducting ATR prism, we have previously shown pH changes at the surface of an electrode modified with iron sulfide under conditions of hydrogen evolution and reduction of carbon dioxide [22]. Here, the approach is demonstrated to be generally applicable to reactions at electrodes modified with a carbon nanotube and carbon nanomaterial. 

## 2. Materials and Methods

**Figure 1:**
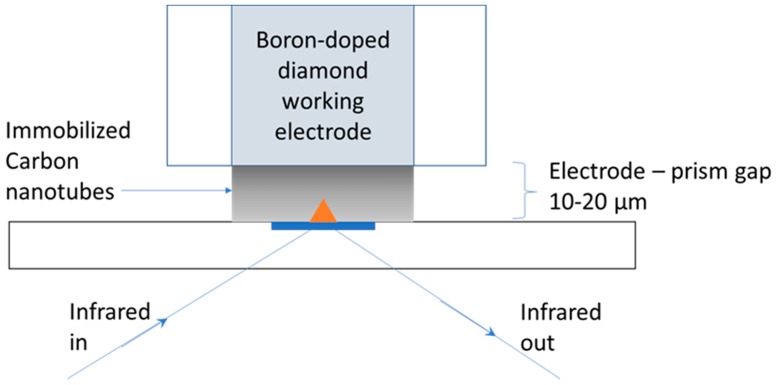
Schematic (not to scale) of the location of the working electrode above the attenuated total reflectance (ATR) prism of the infrared (IR) spectrometer. The orange triangle represents the evanescent wave of the IR radiation’s penetration in the solution, up to 2 µm above the prism. The electrode is immersed in electrolyte, and the cell is completed with counter and reference electrodes (not shown).

A boron-doped diamond working electrode with a diameter of 3 mm was modified with a drop-coated layer of single-walled carbon nanotubes (Sigma Aldrich, Gillingham, Dorset, UK) > 85% carbon > 70% nanotubes; diameter > 2 nm, length < 1 µm). The carbon nanotubes were prepared by adding 0.06 g of the nanotubes to 3 mL of water, followed by vigorous stirring with sonication. An unstable suspension was formed, from which 1.5 µL was immediately drop-coated onto the working electrode, using a pipette, and allowed to dry to form an adherent layer. For each new experiment, the nanotubes were vigorously re-stirred before drop-coating, as they did not remain suspended. A three-electrode cell was constructed above the prism of a Bruker Tensor ATR IR spectrometer, as already described in detail previously [22,23]. The surface of the working electrode modified with a carbon nanotube was located approximately 10–20 µm above the prism surface, as determined by earlier calibration experiments [23]. Upon immersion in the solution, the nanotube layer tended to swell and occupy the region between the electrode surface and the prism, as shown in Figure 1. 

The penetration of the evanescent IR wave approximately 2 µm above the surface of the prism allows the vibrational spectrum of the solvated porous nanotube network just above the prism to be probed. If good electrical contact with the electrode is maintained, changes in the IR spectrum as a function of applied potential can be measured. It is also possible to measure spectral changes using a working electrode that is not modified by carbon nanomaterial (see Section 3.4), but the sensitivity of the measurement decreases, due to the smaller solid–solution interfacial area. The other electrodes in the cell were an Ag/AgCl reference electrode and a Pt coil counter electrode, with the potential of the working electrode being controlled by a potentiostat.

Solutions composed of different ratios of K_2_HPO_4_ and KH_2_PO_4_ (both Sigma Aldrich) were used as electrolytes and pH calibration/reference solutions. KOH or H_3_PO_4_ was also used to increase and decrease the pH, respectively. Milli-Q ultrapure water of 18 MΩ cm was used in all solutions, and the resulting pH was determined using a pH meter. The IR spectra of the phosphate solutions over the pH range 2–14 were measured in the mid-IR region (400–4000 cm^−1^), with a resolution of 4 cm^−1^. The reference (background) spectrum for the measurements was the bare ATR prism, unless noted otherwise. Moreover, both the reference and the sample spectra were obtained from an average of 100 interferograms. As for the in situ spectral recording, while a potential was applied, the software was programmed to record spectra continuously over the duration of the experiment. This was done by using the repeated measurement mode setting in Opus and recording 18 sample spectra, each computed from 15 averaged interferograms. The recording was continuous, i.e., the time interval between each scan was set to 0.

## 3. Results

### 3.1. IR Spectra of Aqueous Phosphate Solutions of Known pH

**Figure 2:**
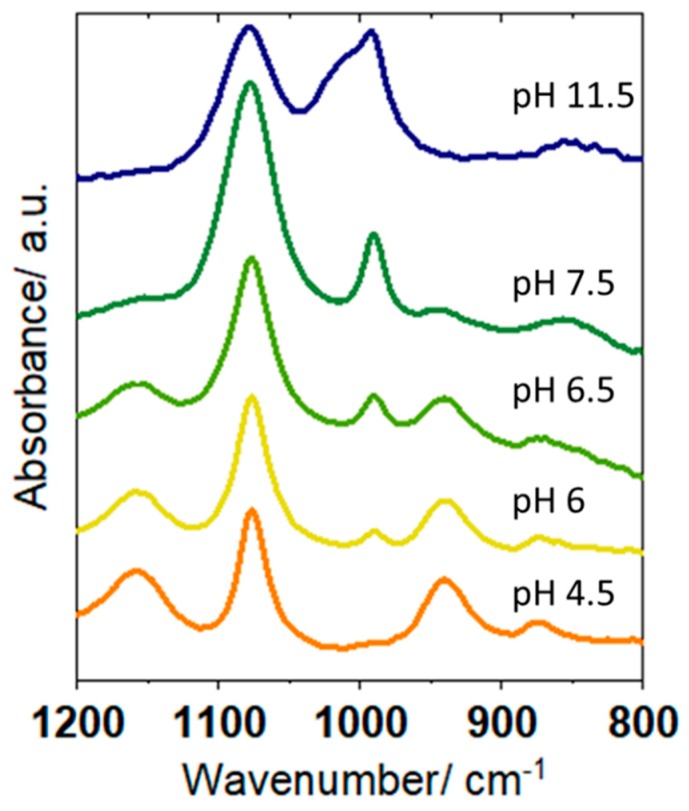
IR spectra of phosphate species in reference solutions, ranging from 800 to 1300 cm^−1^, with pH 6, 6.5, 7.5, and 11.5.

The IR absorbance spectra of solutions of different pH over the range 4.5 to 11.5 with 0.2 to 0.5 intervals were recorded. The selected spectra in the 800 to 1200 cm^−1^ range corresponding to the phosphate bandsare shown in Figure 2. 

The spectra were broadly the same as those reported for these solutions elsewhere [24]. The bands arise due to ν_1_ symmetric stretching and ν_3_ asymmetric stretching vibrational modes in the species present in the phosphate solutions in varying ratios, depending on the pH. The relevant equilibria and pK_a_ values are shown in Equations (1)–(3):

H_3_PO_4_ ↔ H_2_PO_4_^−^ + H^+^   pK_a_^1^ = 2.16 (1)

H_2_PO_4_^−^ ↔ HPO_4_^2−^ + H^+^   pK_a_^2^ = 7.21 (2)

HPO_4_^2−^ ↔ PO_4_^3−^ + H^+^    pK_a_^3^ = 12.32. (3)

Thus, the solution species are H_3_PO_4_ and H_2_PO_4_^−^ at pH near pK_a_^1^; at pH near pK_a_^2^, H_2_PO_4_^−^ and HPO_4_^2−^ dominate, and as the pH approaches pK_a_^3^, the concentration of the PO_4_^3−^ species increases. The IR vibrational bands for the four phosphate species are listed in Table 1, where it can be seen that the symmetry of each species dictates which modes are IR active and where the loss of degeneracy resultsin the splitting of bands.

Figure 2 shows that solutions of pH 4, 6, 6.5, and 7.5 had IR spectra consistent with the existence of H_2_PO_4_^−^ and HPO_4_^2−^ in different ratios. In particular, the H_2_PO_4_^−^ band at 940 cm^−1^ decreased as the pH increased from 6 to 7.5, concomitantly with the increase in the HPO_4_^2−^ peak at 990 cm^−1^. Very small gradations of pH values can therefore be determined from changes in the phosphate IR bands over this pH range. This can be quantified by determining the peak areas for the 990 and 940 cm^−1^ bands using Opus software. These ratios are 0.12 for pH 6, 0.60 for pH 6.5, and 7.20 for pH 7. Similarly, the ratios between the peak areas for the 1078 and 940 cm^−1^ bands can be determined: 1.57 for pH 4.5, 2.71 for pH 6, 5.90 for pH 6.5, and 50.30 for pH 7.5. Increasing the pH still further to 11.5 resulted in a broad increase in absorbance at 1010 cm^−1^, overlaid on the bands for HPO_4_^2−^. This is due to the presence of PO_4_^3−^ in the solution at this pH, as predicted by Equation (3). Similar IR absorbance spectra of phosphate solutions over the whole pH range were recorded (not shown). These acted as reference/calibration spectra, for comparison with in situ spectra measured at the electrode interface. 

### 3.2. Interfacial pH Changes During the Water Reduction Reaction

**Figure 3:**
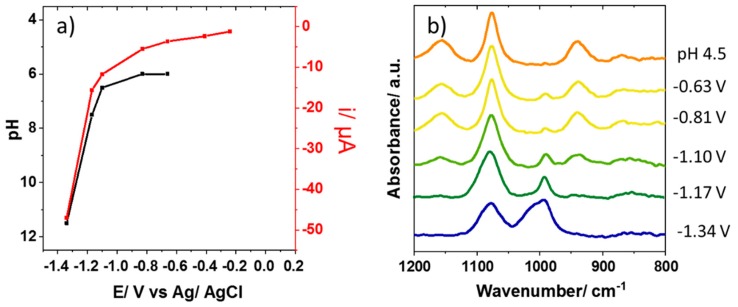
(**a**) Current recorded at an electrode modified with a carbon nanotube at 0.075 M KH_2_PO_4_ (initial pH = 4.5), as a function of applied potential (red) overlaid with the pH of an interfacial solution as a function of potential (black). The current was determined by comparing the recorded IR spectra to the spectra of reference solutions of known pH. (**b**) In situ IR spectra measured at different potentials in a 800–1200 cm^−1^ range.

The IR spectra of the solution in the interfacial region of the electrode modified with a carbon nanotube were recorded as a function of applied electrode potential in 0.075 M KH_2_PO_4_ (initial pH = 4.5). The potential was swept using linear sweep voltammetry, from 0.25 to −1.35 V at 5 mV.s^−1^, while the IR spectra were simultaneously recorded continuously. Figure 3a shows the resulting electrode current plotted as a function of potential (red), overlaid with the pH of the interfacial region at that potential, determined from the IR spectra of the phosphate species (black). To determine these pH values, the in situ IR spectra obtained at the different potentials, shown in Figure 3b, were compared to those of standard phosphate solutions of known pH (e.g., those in Figure 2). For spectra in the pH range 6–7.5, the relative peak areas of the 1078, 990, and 940 cm^−1^ bands can also be determined by comparison to those of the reference spectra. 

The increasing negative currents measured as the applied potential is decreased show that a reduction reaction is taking place. At the pH of the starting solution, this reaction is likely to be a combination of equations (4) and (5):

2H^+^ + 2e^−^ → H_2_ (4)

2H_2_O + 4e^−^ → H_2_ + 2OH^−^. (5)

Both reactions result in an increase in pH due to proton consumption or the generation of hydroxide. At −0.66 and −0.81 V, the IR spectra of the interfacial region (Figure 3b) showed an additional small band at 990 cm^−1^, which is not present in the spectrum of the bulk starting solution. A comparison to Figure 2 shows that the spectra at these potentials were consistent with a solution of pH 6, in contrast to a pH of 4.5 before a potential was applied. The 990 cm^−1^ band can be assigned to the increased concentration of HPO_4_^2−^ resulting from the deprotonation of H_2_PO_4_^−^. At −1.10, −1.17, and −1.34 V, where increased reduction currents were observed, the evolution of the IR spectra was more dramatic. At −1.10 V, the increasing peak at 990 cm^−1^ indicated a further pH increase to 6.5; at −1.17 V, the peak for the H_2_PO_4_^−^ species at 940 cm^−1^ was negligible, and the spectrum was consistent with a pH 7.5 solution. At −1.34 V, the emergence of a band at 1010 cm^−1^ shows that fully deprotonated PO_4_^3−^ was present in the interfacial region, and the spectrum can be matched with the reference for pH 11.5. This shows that the pH change in the region close to the electrode was surprisingly dramatic; a change of 7 pH units was observed, corresponding to a local increase in [OH^−^] from 3 × 10^−10^ to 3 × 10^−3^ mol.dm^−3^. A control experiment, where the electrode was moved several millimeters away from the ATR prism surface, showed no spectral changes under the same conditions, indicating that such pH changes were confined to the region close to the electrode surface. 

### 3.3. Improvement of Sensitivity Using Difference Spectroscopy

As described above, a pH change from 4.5 to 6 was readily discernable from IR spectral changes at a potential of −0.66 V. However, as shown in Figure 3a, some reduction currents were apparent at −0.41 V, for which a pH change has not yet been determined. The difficulty in measuring pH changes under these conditions arises from the composition of phosphate solutions in the pH range 3–5.5, where a relatively large change in pH does not significantly change the ratios of the phosphate species present. For example, at pH 4.5, the solution is composed of 99.5% KH_2_PO_4_ and 0.5% K_2_HPO_4_, while at pH 5.5 the ratio is changed only a little to 95.2% and 4.8%, respectively. In contrast, a more dramatic change, to 86.3% and 13.7%, is found when increasing the pH further by just 0.5, to pH 6. The small changes in H_2_PO_4_^−^ and HPO_4_^2−^ concentrations between pH 4.5 and 5.5 mean that the spectral signatures of these solutions are almost impossible to tell apart, especially given the overlapping wavenumber positions of the key bands (Table 1). 

**Figure 4:**
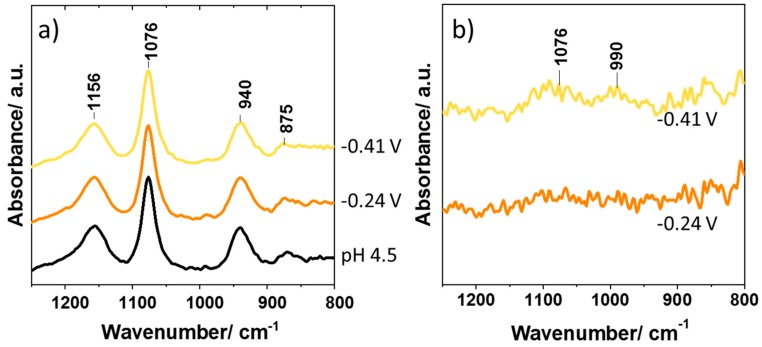
(**a**) Absorption spectra measured at −0.24 and −0.41 V, along with a spectrum of 0.075 M KH_2_PO_4_ and a starting solution of pH 4.5 (air as background); (**b**) Difference spectra recorded at −0.24 and −0.41 V, using a spectrum of electrode-immersed 0.075 M KH_2_PO_4_ as the background reference spectrum.

Figure 4a shows the in situ IR absorbance spectra at the electrode interface at potentials of −0.24 and −0.41 V, where changes between the spectra and that of the starting pH 4.5 solution are not very apparent. However, if the spectrum of the electrode interface immersed in pH 4.5 KH_2_PO_4_ without applied potential is measured and used as a background spectrum, the corresponding difference spectra can be obtained. Figure 4b shows the spectrum obtained at −0.24 V using the spectrum under the same conditions but with no applied potential as the background. The spectrum is largely featureless, indicating no change in the composition of the solution when applying −0.24 V. However, at −0.41 V, features at ca. 1076 and 990 cm^−1^ begin to emerge, indicating an increased concentration of HPO_4_^2−^ at this potential, consistent with an increase in pH associated with the onset of water/proton reduction (Equations (4) and (5)). Although these measurements are yet to be optimized (longer spectral acquisition times are needed to improve the signal-to-noise ratio), we have hereby shown that it may be possible to observe small changes in the composition ofsolutions by using this technique. What is required is a simple consideration of suitable reference spectra. However, this method must be further refined, to ensure that the observed spectral features do not result from background artifacts.

### 3.4. In Situ Determination of pH Change During the Oxidation of Hydroquinone

The suitability of this technique for the in situ monitoring of pH changes in the electrode diffusion layer was further investigated with regard to the oxidation of hydroquinone (1,4-benzenediol, H_2_Q) to quinone (1,4-benzoquinone, Q) (Equation (6). 

H_2_Q ↔ Q + 2H^+^ + 2e^−^ (6)

This reaction yields 2 mols of protons per mol of oxidized H_2_Q. Accordingly, the pH in the diffusion layer is expected to decrease as the reaction proceeds. Initial cyclic voltammetry experiments showed that the electrode modified with carbon nanotubes suffered adsorption effects, which prevented a clear observation of the redox events and potentials. Therefore, a bare boron-doped diamond electrode was used, at which the 5 × 10^−3^ mol.dm^−3^ H_2_Q underwent oxidation at E_p_ = 0.4 V vs. Ag/AgCl in a 0.075 mol.dm^−3^ phosphate buffer solution with a pH of 6.8. The same electrode was used for the in situ spectroelectrochemistry measurements, and a potential of 0.55 V was applied to ensure that H_2_Q was oxidized under diffusion control. 

**Figure 5:**
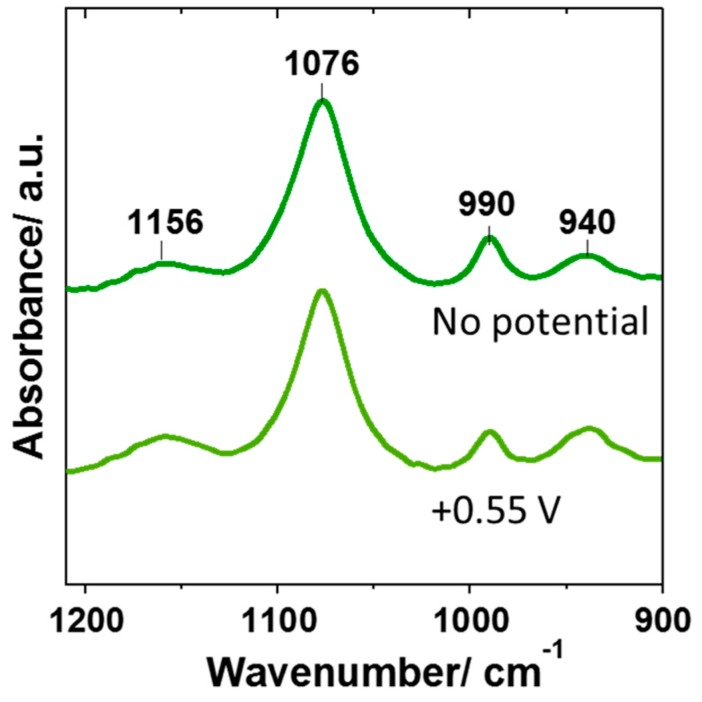
In situ IR spectra in the electrode interface before (top) and after (bottom) the application of 0.55 V to a 0.075 mol.dm^−3^ phosphate buffer solution with 5 × 10^−3^ mol.dm^−3^ H_2_Q and a pH of 6.8.

Figure 5 shows the IR spectra of the phosphate bands in the interfacial region before and after the potential was applied. Initially, bands for both H_2_PO_4_^−^ and HPO_4_^2−^ species can be observed, consistent with those expected for the 0.075 mol.dm^−3^ phosphate buffer solution with a pH of 6.8. When applying 0.55 V, the same bands were still present, but the 990 cm^−1^ band corresponding to HPO_4_^2−^ became less intense, while the H_2_PO_4_^−^ band at 940 cm^−1^ increased. This is consistent with a decrease in pH (as more protons are present), and a comparison with reference spectra shows that the pH under these conditions was 6.5. A control experiment where a potential of 0.55 V was applied in the absence of H_2_Q showed no changes in the IR spectrum, confirming that the pH change was due to the H_2_Q oxidation reaction. 

The observed change in pH represents a local increase in proton concentration in the IR sampling region from 1.6 × 10^−7^ (pH 6.8) to 3.2 × 10^−7^ mol.dm^−3^ (pH 6.5). Considering the stoichiometry of Equation (6), this increase is smaller than expected. The complete oxidation of a 5 × 10^−3^ mol.dm^−3^ solution of H_2_Q is expected to yield 1 × 10^−2^ mol.dm^−3^ of protons, which, if confined entirely to the interfacial region between the electrode and the ATR prism, would result in a measured pH of 2. However, the sampling region is not an entirely confined space, especially when the electrode is bare and unmodified with carbon nanotubes. The electrode to prism separation has previously been determined as up to 20 µm, allowing solution species to diffuse in and out of the gap according to a concentration gradient. Thus, the HPO_4_^2−^ species that are lost during protonation are rapidly replaced by the flux of new HPO_4_^2−^ into the electrode diffusion layer. Conversely, the H_2_PO_4_^−^ species formed through protonation rapidly diffuse away from the electrode interface into the bulk solution. The results therefore suggest that buffering of the pH near the electrode is very efficientunder these experimental conditions.

To investigate the effectiveness of the buffering process as the phosphate concentration decreases, the experiments were repeated with the same 5 × 10^−3^ mol.dm^−3^ concentration of H_2_Q, but with phosphate concentrations of 0.1, 0.05, and 0.025 mol.dm^−3^. The pH of the initial solution remained 6.8, and the ionic strength was maintained at 0.3 mol.dm^−3^ throughout the addition of KCl. The observed pH, determined by the IR spectrum, is shown in Table 2, and the change in pH is clearly more pronounced as the concentration of phosphate species decreases. This is due to the decreased mass transport of HPO_4_^2−^ and H_2_PO_4_^−^ into and out of the interfacial region at lower concentrations, as the concentration gradients—and hence the flux—decrease. The solution is thus less able, at lower concentrations, to buffer the pH changes. These observations illustrate the importance of phosphate concentration in maintaining a constant pH near the electrode during buffering. 

## 4. Discussion and Conclusions

The aim of this work was to demonstrate the applicability of in situ IR spectroscopy to measure changes in pH in electrode surfaces modified with carbon nanomaterial while a potential is applied. Previously reported methods to measure pH at electrodes lack the versatility required to study complex nanomaterials over a sufficiently wide pH range. This method is based on a simple drop-coating of the working electrode with the material under investigation. The modified electrode is then immersed in a solution over the ATR prism and IR spectra measured while a potential is applied. The method relies on the presence of “reporter ions” such as phosphate, whose pH-dependent equilibria allow for the determination of pH based on the ratio of differently protonated species in solution. Other species such as acetate, borate, or perchlorate could also be used if different solution conditions or pH ranges were of interest. We have shown that pH changes can be determined for key reactions such as water reduction (hydrogen evolution) and the proton-coupled oxidation of hydroquinone. 

The role of mass transport (diffusion) of phosphate species in effective buffering of pH was also shown. The cell setup in this method inevitably leads to a more confined interfacial region, which may not be representative of all electrode geometries and applications. However, the buffering ability of the electrolyte shows that diffusion is not hindered to a problematic degree. The confined interfacial region may also be a better model for the electrode surface structure in some key electrochemical applications requiring high surface carbon nanomaterials, for example in supercapacitors, capacitive deionization, and embedded electrodes in biological sensing. 

In conclusion, we have shown that pH changes of many orders of magnitude can take place at carbon electrodes during the electrochemical reduction of water. Smaller pH changes can also be detected in connection with proton-coupled electron transfers. The in situ IR technique appears to be a flexible, quick, and non-invasive means of obtaining information about the composition of the electrode interface during operation. 

## Figures and Tables

**Table 1:** Wavenumbers of main IR vibrational bands for phosphate species in an aqueous solution.

Species	Wavenumber/cm^−1^ ν_3_	ν_3_	ν_3_	ν_1_
H_3_PO_4_	1172	1005		889
H_2_PO_4_^−^	1159	1077	940	875
HPO_4_^2−^	1078	990		850
PO_4_^3−^	1010			

**Table 2:** Observed pH values in the electrode interface at 0.55 V, in presence of 5 × 10^−3^ mol.dm^−3^ H_2_Q with different concentrations of phosphate (initial pH = 6.8, ionic strength = 0.3 mol.dm^−3^).

Phosphate Concentration/mol.dm^−3^	pH	[H^+^]/mol.dm^−3^
0.100	6.6	2.5 × 10^−7^
0.075	6.5	3.2 × 10^−7^
0.050	6.4	4.0 × 10^−7^
0.025	6.1	7.9 × 10^−7^
